# Re: “Seroprevalence of Borrelia IgM and IgG Antibodies in Healthy Individuals: A Caution Against Serology Misinterpretations and Unnecessary Antibiotic Treatments” by Strizova et al.

**DOI:** 10.1089/vbz.2020.2663

**Published:** 2020-09-29

**Authors:** Miriam F. Weiss

**Affiliations:** Department of Bioethics, Case Western Reserve University School of Medicine, Cleveland, Ohio, USA.

Strizova et al.'s (2020) recent review article focuses on the inaccuracy of serologic diagnosis in Lyme disease. We agree that serologic testing alone is not sufficient for diagnosis. However, we wish to call your readers' attention to research that suggests persistent positivity for IgM antibodies is an important sign of chronic infection by the Lyme spirochete.

*Borrelia burgdorferi* has been shown to manipulate both the innate and adaptive immunity of its mammalian hosts in a variety of ways (Tracy and Baumgarth 2017). Early in infection, it migrates to the lymph nodes (Tunev et al. 2011). There it interferes with the maturation of memory B cells, and causes the failure to develop long-term immunity (Elsner et al. 2015).

In short, the persistence of IgM antibodies in Lyme disease may represent chronicity, not a false positive. The classical pattern of B cell maturation and the IgM to IgG transition are lacking in chronic infection by *Borrelia burgdorferi*. The organism abrogates the development of highly specific long-term immunity to persist in the host (Elsner et al. 2015).
